# Anaphylactic Shock During Pediatric Anesthesia: An Unexpected Reaction to Sevoflurane

**DOI:** 10.3389/fped.2018.00236

**Published:** 2018-09-07

**Authors:** Alessandro Simonini, Etrusca Brogi, Brunella Gily, Mariangela Tosca, Claudia Barbieri, Francesca Antonini, Genny Del Zotto

**Affiliations:** ^1^Department of Anaesthesia and Intensive Care, Istituto Giannina Gaslini, Genova, Italy; ^2^Department of Anesthesia and Intensive Care, University of Pisa, Pisa, Italy; ^3^Department of Anaesthesia and Intensive Care, University of Genova, Genova, Italy; ^4^Allergy Unit, Department of Pediatrics, Istituto Giannina Gaslini, Genova, Italy; ^5^Department of Nursing and Health Professionals, Istituto Giannina Gaslini, Genova, Italy; ^6^Department of Research and Diagnostics, Istituto G. Gaslini, Genoa, Italy

**Keywords:** Sevoflurane, anaphylaxis, anaphylactic shock, allergy, perioperative complications, pediatric anesthesia

## Abstract

During general anesthesia, while muscle relaxants, latex and antibiotics are normally considered as very common causes of anaphylactic reactions, there are no documented cases of anaphylaxis due to inhalational agents. We report the case of a 6-year-old child scheduled for adenotonsillectomy who had an anaphylactic shock reaction due to Sevoflurane. Several allergic tests were performed to detect the trigger. Drugs used during operation were tested on both patient and three matched controls. While controls were negative, the patient displayed a positive reaction to Sevoflurane. To our knowledge, this is the first published report describing an allergic reaction caused by a volatile anesthetic.

## Introduction

Anaphylaxis is a severe, potentially life-threatening systemic reaction (1) and represents one of the most fearsome emergencies in the perioperative period. Data on pediatric anaphylaxis are poor; the few studies published over the last years reported incidence rates ranging from 0.11 to 0.41% (2), with a mortality rate of 3–9% (3, 4).

Since anaphylaxis is a rapidly evolving emergency, immediate management must be based on prompt diagnosis and appropriate treatment (5). In 2009, the Association of Anesthetists of Great Britain and Ireland published guidelines on suspected anaphylactic reactions associated with anesthesia, recommending to carry out preoperative diagnostic investigations (6). Still too often, however, patients are not investigated appropriately (7).

All drugs and surgery-associated agents (i.e., Chlorhexidine, latex) used intraoperatively can potentially cause allergic reactions and the prompt detection of a potential allergen is compulsory. Muscle relaxants are the most common causes, followed by latex and antibiotics. By contrast, anaphylaxis due to opioids is very rare (8, 9). Noteworthy, there are no documented cases of inhalational agents causing anaphylaxis.

Here we report the case of a pediatric patient scheduled for adenotonsillectomy, who had an anaphylactic shock due to Sevoflurane. To our knowledge, this is the first report describing an allergic reaction caused by a volatile anesthetic.

### Case report

A 6-year-old female child (114 cm, 17 kg) scheduled for adenotonsillectomy. Due to a traumatic fracture, she wore a plaster cast in the upper limb. She reported a history of sleep apneas. Notably, her past medical history did not include any reaction to drugs or food. Pre-operative physical examination was negative.

On the day of surgery, preoxygenation was applied and anesthesia was induced with Sevoflurane at increasing concentrations. Baseline oxygen saturation, non-invasive blood pressure (NIBP) and heart rate were 98%, 106/57 mmHg and 112 beats/min, respectively. A 22-gauge cannula was inserted and Fentanyl (1 μg/kg), Propofol (3 mg/kg), Dexamethasone (0.3 mg/kg) and Rocuronium (0.6 mg/kg) were administered.

The patient underwent oral intubation (4.5-mm cuffed endotracheal tube) without complications. Anesthesia was maintained with Sevoflurane (Minimum Alveolar Concentration 1, MAC 1) and Remifentanil (0.25 μg/kg/min). Mechanical ventilation was started with low tidal volume (6–7 ml/kg), a positive-end expiratory pressure setting of 5 cm H_2_O, and a FiO_2_ of 0.3.

After 7 min from initial drug administration, we observed a collapse of NIBP (58/17 mmHg), of SpO_2_ (to 77%) and a decrease of EtCO_2_ (to 26 mmHg). Overall, there was a decrease of more than 30% from baseline. The procedure was suddenly suspended together with Remifentanil infusion, while Sevoflurane was maintained for neuroprotection and awareness prevention. Initial resuscitation with a prompt infusion of saline solution (20 mL/kg) and Albumin 5% (i.e., 15 mL/kg), did not improve clinical parameters. We also observed a continuous worsening of SpO_2_ and an increase in peak pressure (up to 43 cmH_2_O). A quick check of the orotracheal tube position was performed, immediately followed by endotracheal suction maneuver and an increase in FiO_2_ (up to 1). SpO2 slightly increased (to 92%) without, however, any improvement in NIBP. Consequently, adrenaline was administered (two bolus of 20 mcg each) with a quick but transient improvement of NIBP value. Dopamine infusion was started (from 4 μg/kg/min up to 12 μg/kg/min) without any effect. Femoral arterial and central venous catheters were inserted. Due to the persistence of hypotension, Adrenaline was continuously administered (i.e., 0.4 μg/kg/min), followed by the administration of Hydrocortisone (5 mg/kg) while Sevoflurane was suspended only when an hemodynamic stability (due to Adrenaline infusion) was reached. At this point, we observed a progressive improvement of NIBP (up to 119/55 mmHg) and SpO2 (up to 100%).

In order to reach a precise diagnosis, we performed hemogasanalysis, thoracic X-Ray and echocardiography examinations, which excluded potential cardiovascular and respiratory diseases, as for example pneumothorax, pulmonary embolism, cardiogenic shock). Consequently, we hypothesized an anaphylactic shock. Tryptase test was positive (12, range 1–10) whereas total IgE test (PRIST, Paper Radio Immuno-Sorbent Test) was negative.

Finally, the patient was extubated in the operating room without the need of reversal agents then she was transferred to the PICU (Pediatric Intensive Care Unit) for monitoring and after 24 h she was discharged. Neither neurological nor systemic impairments were reported.

## Materials and methods

A written informed consent to publish all the data concerning this case report was obtained from the patient's parents.

### Basophil activation test (BAT)

BAT was performed twice, namely 3 days and 4 weeks after surgery. Peripheral blood (PB) samples were obtained from our patient and three matched controls and collected in EDTA (ethylendiamintetraacetic acid)-containing blood collection tubes. In order to study both the IgE-independent and IgE-dependent activation pathways, basophil reactivity to stimulation was evaluated with two different stimuli, fMLP and an anti-IgE monoclonal antibody, respectively. Flow CAST (Bühlmann, Schönenbuch, Switzerland) reagents were used and basophils were treated following manufacturer's instructions. In brief, 100 μl of IL-3 containing stimulation buffer was added to 50 μl of peripheral blood in each analysis tube. Subsequently, 20 μl of anti-CCR3 PE and anti-CD63 FITC were added to each tube. To test Sevoflurane, which at room temperature and pressure is found in a gaseous state, we added the drug, closed each tube tightly and vortexed. To test the other drugs (i.e., Rocuronium, Sugammadex, Fentanyl, Remifentanil, and Propofol) we added 50 μl of each of them at therapeutic concentration plus two scalar dilutions (1:10 and 1:100, see Table 1) in each tube of the patient and matched controls. Cells were then incubated in a 37°C water bath for 15 min. Afterwards, samples were lysed for 8 min and then centrifuged at 1,750 r.p.m. for 5 min. Finally, cells were resuspended with 400 μl of washing buffer and evaluated by flow cytometry (Gallios, Beckman Coulter Inc., Brea, CA). Resulting fsc files were then analyzed with Kaluza software (Beckman Coulter Inc., Brea, CA). SSC^low^ CCR3^+^ cells were considered as basophils (10) and their activation was evaluated by means of their CD63 surface expression, CD63^+^ basophils were considered activated (11). Cut off values used to verify basophil functionality were those recommended by the manufacturer.

**Table 1:** Analyzed drugs and dilutions used.

**Drug (therapeutic concentration)**	**Dilution**	**Dilution**	**Dilution**
Rocuronium (5 mg/ml)	1:1	1:100	1:1,000
Sugammadex (10 mg/ml)	1:1	1:100	1:1,000
Rocuronium + Sugammadex (5 mg/ml + 10 mg/ml)	1:1	1:100	1:1,000
Fentanyl (10 ug/ml)	1:1	1:100	1:1,000
Remifentanil (20 ug/ml)	1:1	1:100	1:1,000
Propofol (10 mg/ml)	1:1	1:100	1:1,000
Ketamine (10 mg/ml)	1:1	1:100	1:1,000
Midazolam (1 mg/ml)	1:1	1:100	1:1,000
Sevoflurane (gaseous)	Not applicable	Not applicable	Not applicable
Chlorhexidine 5 mg/ml in ethanol 70%	1:100	1:1,000	1:10,000
Antisapril (5 ul/1 ml di H_2_O)	1:100	1:1,000	1:10,000

## Results

Considering that commercial standardized reagents to test the above mentioned drugs were not available on the market, in order to avoid false positive results, we always checked every single drug (in all three dilutions) not only on our patient but also on three healthy matched controls. Moreover, following allergist's suggestion, we also tested other drugs as possible options in case of further surgery.

**Figure 1:**
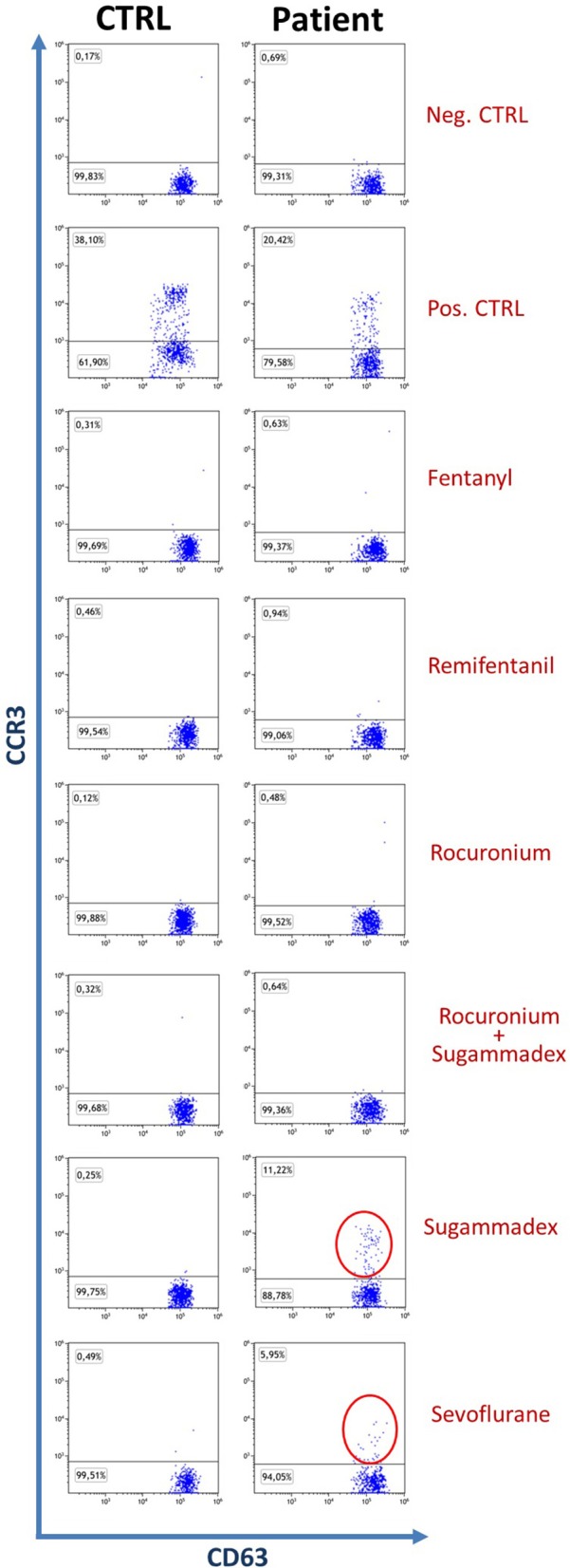
Dot plot of patient and control basophils (SSC^low^CCR3^+^CD63^+^), the control shown is representative of three. The percentage of patient positivities is highlighted with red circles.

At first, we analyzed basal basophil reactivity and we verified that basophils of all four subjects were not activated under resting conditions (the percentage of CD63^+^ basophils was below 1% in all of them) and that they were all able to undergo degranulation upon stimulation with fMLP or anti-IgE (the percentage of CD63^+^ basophils was far above 10% in all subjects). After this, we stimulated the PB of all our subjects with the drugs of interest. Based on manufacturer's indications, samples are considered positive for drugs when more than 5% of basophils were activated. In our experiments, all controls turned out to be negative while the patient displayed a positive reaction to Sugammadex (11.22%) and Sevoflurane (5.95%), as shown in Figure 1. Further investigations, performed with Prick test and intradermalreaction (12), confirmed Basophil Activation Test results.

Prick test and intradermalreaction also showed hypersensitivity to Midazolam and Mivacurium, identifying the child as a patient at high anesthetic risk.

## Discussion

In the suspicion of anaphylactic reaction it is important to perform plasma tryptase and total IgE tests, both being useful to confirm the diagnosis. In the postoperative period, a clinical and laboratory allergy evaluation is compulsory. Furthermore, all the substances/drugs with which the patient has come into contact should be tested and results confirmed (13) by different methods (BAT, Prick, Patch tests etc.). The tests should be performed twice, 3–4 days and 4–6 weeks after the allergic reaction (14). As already mentioned, in order to provide the patient with safe alternatives for possible future anesthetic treatments, we think that allergic tests should be performed on a wide range of anesthetic agents. A recent survey showed that the anesthetics responsible for anaphylactic reactions are, in order of incidence, NMBA (38.5%), antibiotics (38.3%), contrast media (6.7%), Chlorhexidine (3.9%), analgesics (3.3%), intravenous fluids including colloids (2.8%), latex (1.5%), Inducers (0.9%), antiemetics (0.9%), hemoderivatives (0.6%), reversal agent (0.5%) and local anesthetics (0.5%) (15). We took advantage of this survey to choose which substances had to be tested. We focused our attention on some analgesics (Fentanyl, Remifentanyl), inducers (Propofol, Midazolam and Ketamine), on NMBAs (Rocuronium, Mivacurium) and their antagonists (Sugammadex), and also on decontaminants used in our operating room (Chlorhexidine and Antisapril) (see Table 1). Together with positivity to Sugammadex and Sevoflurane, our laboratory investigations brought to light a patient hypersensitivity to midazolam and mivacurium, identifying the child as a patient at high anesthetic risk and suggesting the need, in case of future anesthesiological interventions, to administer a prophylaxis with antihistamines and steroids.

To our knowledge, anaphylactic reactions to halogenated drugs have not been described in the literature (8) yet. Our case report take home message is to remember that, in case of allergic events, also volatile anesthetics can be responsible of anaphylactic reactions, and sometimes they should be promptly suspended. Furthermore, after an allergic reaction, we strongly encourage anesthetists to request appropriate blood tests to recognize the responsible drug and to help build more refined statistics of anesthesiologic anaphylaxis that better matches the reality of these severe complications.

## Author contributions

AS, BG, and CB clinically managed the patient. GD and FA performed and analyzed the cytometric allergic tests. MT followed the patient allergic outcome. AS, EB, and GD wrote the paper. AS and GD coordinated all clinical, laboratory and scientific works.

### Conflict of interest statement

The authors declare that the research was conducted in the absence of any commercial or financial relationships that could be construed as a potential conflict of interest.
